# Enhancing Pediatric Adnexal Torsion Diagnosis: Prediction Method Utilizing Machine Learning Techniques

**DOI:** 10.3390/children10101612

**Published:** 2023-09-27

**Authors:** Ahmad Turki, Enas Raml

**Affiliations:** 1 Electrical and Computer Engineering Department, Faculty of Engineering, King Abdulaziz University, Jeddah 21589, Saudi Arabia; 2 Center of Excellence in Intelligent Engineering Systems, King Abdulaziz University, Jeddah 21589, Saudi Arabia; 3 Pediatric Surgery Department, Faculty of Medicine, King Abdulaziz University, Jeddah 21589, Saudi Arabia; enas.ramel@gmail.com; 4 Pediatric Surgery Department, International Medical Center, Jeddah 23214, Saudi Arabia

**Keywords:** pediatric adnexal torsion, acute appendicitis, support vector machine, machine learning, diagnosis, clinical manifestations

## Abstract

This study systematically examines pediatric adnexal torsion, proposing a diagnostic approach using machine learning techniques to distinguish it from acute appendicitis. Our retrospective analysis involved 41 female pediatric patients divided into two groups: 21 with adnexal torsion (group 1) and 20 with acute appendicitis (group 2). In group 1, the average age was 10 ± 2.6 years, while in group 2, it was 9.8 ± 21.9 years. Our analysis found no statistically significant age distinctions between these two groups. Despite acute lower abdominal pain being a common factor, group 1 displayed shorter pain duration (28.9 h vs. 46.8 h, *p* < 0.05), less vomiting (28% vs. 50%, *p* < 0.05), lower fever incidence (4.7% vs. 50%, *p* < 0.05), reduced leukocytosis (57% vs. 75%, *p* < 0.05), and CRP elevation (30% vs. 80%, *p* < 0.05) compared to group 2. Machine learning techniques, specifically support vector classifiers, were employed using clinical presentation, pain duration, white blood cell counts, and ultrasound findings as features. The classifier consistently demonstrated an average predictive accuracy of 87% to 97% in distinguishing adnexal torsion from appendicitis, as confirmed across various SVM models employing different kernels. Our findings emphasize the capacity of support vector machines (SVMs) and machine learning as a whole to augment diagnostic accuracy when distinguishing between adnexal torsion and acute appendicitis. Nevertheless, it is imperative to validate these results through more extensive investigations and explore alternative machine learning models for a comprehensive understanding of their diagnostic capabilities.

## 1. Introduction

Adnexal torsion involves the twisting of the fallopian tube and/or ovary with its vascular pedicle [1]. Early suspicion and diagnosis are crucial to prevent damage and necrosis of the ovaries [2].

Adnexal torsion is considered a rare cause of acute abdominal pain in children and adolescents, with an incidence of 2 to 4.9 cases per 100,000 individuals [1], peaking between the ages of 9 and 14 years [2].

Other conditions, such as ovarian cysts or acute appendicitis, can mimic the symptoms of adnexal torsion, complicating the diagnosis [3]. Moreover, mesenteric adenitis, gastroenteritis, urinary tract infections, and renal stones can likewise present with acute onset abdominal pain and should be considered in the differential diagnosis.

Ultrasound imaging is typically the initial step in evaluating suspected cases of adnexal torsion. It helps assess blood flow to the ovaries and identifies signs of torsion such as enlarged ovaries, twisted blood vessels, or a twisted pedicle [1].

Although ultrasound or Doppler flow are valuable tools for diagnosing adnexal torsion, they may not always provide a definitive diagnosis on their own [4]. Additional diagnostic methods and clinical evaluation may be required to confirm the condition [3].

If adnexal torsion is strongly suspected but not confirmed by ultrasound, prompt surgical intervention may be necessary to prevent further complications [5]. Laparoscopic exploration is often performed to definitively diagnose adnexal torsion and determine the appropriate treatment, which may involve untwisting the affected structures (detorsion), ovarian fixation, or excision of the ovarian cyst if needed [5].

Several studies emphasize the importance of improving the time to diagnose adnexal torsion and its accuracy, utilizing clinical, laboratory, and imaging characteristics [6,7,8]. Rapid diagnosis is essential for successful outcomes, particularly when detorsion, while preserving the ovary, is desired [6]. A recent investigation has revealed that, despite the availability of diagnostic methods, there are instances where this condition goes unnoticed. Consequently, late diagnosis results in surgical procedures such as oophorectomy or adnexectomy being conducted in roughly half of adnexal/ovarian torsion cases [9].

The rise in the adoption of Medical Decision Support Systems (MDSSs) has led to a widespread presence of diagnostic tools aimed at aiding medical practitioners in making precise and effective diagnoses. These systems have substantial promise in enhancing decision-making accuracy for surgeons, physicians, and healthcare professionals, ultimately lowering the risk of errors in critical medical contexts. In pursuit of this goal, MDSS commonly utilizes a variety of machine learning algorithms to streamline the decision-making procedure [10].

Machine learning stands out as a significant and extensively applied field within artificial intelligence. It concentrates on creating techniques and algorithms that empower computers and systems to learn from data and independently execute a range of tasks. Its applications span diverse areas, including data mining, statistics, pattern recognition, data classification, and bioinformatics, among others [11].

Incorporating sophisticated mathematical and statistical software, such as support vector machines (SVMs), into these systems enables them to predict and classify different potential outcomes. Moreover, these advanced systems facilitate faster and more in-depth analysis of vast medical databases, leading to more informed decisions.

The primary outcome of this study is to enhance the diagnostic accuracy of pediatric adnexal torsion using machine learning techniques. Secondary outcomes are to evaluate the clinical manifestations of adnexal torsion, and laboratory and ultrasound findings in pediatric patients, and differentiate between adnexal torsion and acute appendicitis.

## 2. Support Vector Machine

A support vector machine (SVM) is a supervised machine learning algorithm that seeks a linear model that maximizes the margin between hyperplanes for class separation. Data points closest to this margin are called support vectors and are crucial for boundary definition. SVM employs quadratic programming for linearly separable data, and kernel functions for non-linearly separable data, mapping the data into higher-dimensional spaces. Kernel functions include linear, polynomial, and radial basis functions (RBFs). The kernel trick transforms data points to establish optimal decision boundaries, enabling efficient handling of high-dimensional data. SVM finds applications in image classification, text categorization, bioinformatics, and medical diagnosis. Its success depends on kernel function selection and hyperparameter tuning [11,12,13].

## 3. Methodology

We conducted a retrospective study involving female pediatric patients under the age of 14 years, who had undergone surgery for either acute adnexal torsion (group 1) or acute appendicitis (group 2) and were admitted to the emergency department within the same timeframe. This study was conducted at the International Medical Center in Jeddah, Saudi Arabia, between June 2017 and June 2021. To optimize the training data for the SVM classifier aimed at diagnosing adnexal torsion, we intentionally selected a larger number of patients for group 1 compared to group 2. We included a total of 21 patients in group 1 and 20 patients in group 2 based on the following specific inclusion and exclusion criteria.

Inclusion criteria for group 1 (adnexal torsion):

1. Pediatric age less than 14 years
2. Primary adnexal torsion (torsion without adnexal pathology)
3. Secondary adnexal torsion (torsion due to adnexal pathology)

Exclusion criteria for group 1 (adnexal torsion):

1. Neonatal adnexal torsion age less than 1 month
2. Age more than 14 years
3. Adnexal mass without torsion

Inclusion criteria for group 2 (acute appendicitis):

1. Female patient presented with acute appendicitis.
2. Age less than 14 years
3. Duration of pain less than 5 days

Exclusion criteria for group 2 (acute appendicitis):

1. Age more than 14 years
2. Male patients with acute appendicitis
3. Appendicular mass or abscess
4. Patients who present with diffuse peritonitis
5. Duration of pain more than 5 days

The selection of features for our model was guided by the aim to effectively distinguish between pediatric adnexal torsion and appendicitis, both of which can present with similar symptoms. We chose four specific features based on their clinical relevance and their potential to differentiate between the two conditions in our specific patient population.

- Clinical Presentation: Pain and vomiting were chosen because they are common symptoms in both groups.
- Pain Duration: This feature helps capture the temporal aspects of the patients’ pain.
- White Blood Cell (WBC) Count: Elevated WBC counts are a crucial marker of inflammation and infection, which can vary between adnexal torsion and appendicitis cases. This feature contributes to the diagnostic process by considering the inflammatory response in these conditions.
- Radiologic Findings: Ultrasound findings were included as they provide valuable imaging data specific to each condition.

These four features were selected to comprehensively capture both clinical and radiological aspects of the patients’ presentation, enabling our machine learning model to make accurate distinctions between the two groups.

The primary outcome of this study aimed to enhance the diagnostic accuracy of pediatric adnexal torsion using machine learning techniques. Secondary outcomes are to evaluate the clinical manifestations of adnexal torsion, and laboratory and ultrasound findings, in pediatric patients and differentiate between adnexal torsion and acute appendicitis.

To accomplish this, we retrospectively reviewed cases of adnexal torsion and appendicitis, both confirmed through surgical and pathological records. The identified cases were then used to train and test a support vector machine (SVM) machine learning model. We utilized four key clinical features to build the SVM classification model: clinical presentation, pain duration, white blood cell (WBC) counts, and ultrasound findings. These features were converted into numerical values for data vectorization. The assigned numerical values for clinical presentation and ultrasound findings can be found in Table 1 and Table 2, respectively. These values were meticulously determined to accurately represent the corresponding clinical indications and ultrasound results.

To identify the most effective feature for aiding in the diagnosis and confirmation of adnexal torsion, we divided the dataset into two subsets: approximately 20% for training and the remaining 80% for testing. Additionally, we applied *t*-tests to identify the statistically significant features distinguishing the two groups. We evaluated the performance of the SVM model using metrics including accuracy, area under the receiver operator curve (AUC), precision score, recall score, and F1 score, by using the Equations (1)–(5) as shown below. To ensure robust assessment of these metrics, we employed cross-validation for each classifier. Given the relatively limited size of our dataset, this step was vital to mitigate potential biases caused by overfitting.

The SVM approach was implemented with various kernel functions, namely the linear kernel function, polynomial function, and radial basis function. This involved establishing a hyperplane using the training dataset and subsequently applying the trained model to the testing dataset. The constructed hyperplane aided in classifying whether individuals had adnexal torsion.

The accuracy of the SVM metrics was calculated using appropriate equations for evaluation across the three kernel functions. The metrics were determined by considering True Positive (TP), True Negative (TN), False Positive (FP), and False Negative (FN) cases, where TP signifies accurate prediction of individuals with torsion, TN represents accurate prediction of individuals without torsion, FP denotes incorrect prediction of individuals with torsion (Type I error), and FN indicates incorrect prediction of individuals without torsion (Type II error).

All the SVM testing and statistical analyses were conducted using Python (version 3.11; Python Software Foundation, Wilmington, DE, USA).

(1) $$\text{Accuracy}=\frac{\left(TP+TN\right)}{\left(TP+TN+FP+FN\right)}\times 100$$

(2) $$\text{AUC}=\int_{0}^{1}\text{ROC}\;\text{curve}\left(TP\right)\;d\left(FP\right)\times 100$$

(3) $$\text{Precision}=\frac{TP}{\left(TP+FP\right)}\times 100$$

(4) $$\text{Recall}=\frac{TP}{\left(TP+FN\right)}\times 100$$

(5) $$F1\;\text{Score}=2\times \frac{\text{Precision}\times \text{Recall}}{\left(\text{Precision}+\text{Recall}\right)}\times 100$$

Furthermore, we conducted a comparative analysis of the SVM results with those obtained from logistic regression and XGBoost models, aiming to assess the performance and suitability of these machine learning techniques for our specific diagnostic task. Additionally, we utilized the power of SHAP values (SHapley Additive exPlanations) which are a robust tool to understand how each feature contributes to predicting model results [14].

The computation of SHAP values is rooted in the idea that each prediction generated by a machine learning model can be understood as the result of collaborative contributions from the model’s features. In this context, each feature makes a distinct contribution to the prediction’s final outcome. SHAP’s core objective is to fairly allocate the “credit” or “responsibility” for the prediction among these individual features [14].

The SHAP equation is defined as follows:

(6) $$\phi \left(f\right)=\phi_{0}+\sum_{j=1}^{M}\frac{f\left(x_{i}\right)-E\left[f\left(x_{i}\right)\right]}{M}$$

where:

- $\phi \left(f\right)$ represents the SHAP values for a particular feature.
- $\phi_{0}$ is the expected value of the model’s prediction.
- *M* is the total number of features.
- $f\left(x_{i}\right)$ is the model’s prediction when feature *i* is included.
- $E\left[f\left(x_{i}\right)\right]$ is the expected prediction when feature *i* is excluded.

In the context of “Adnexal Torsion” and “Appendicitis”, SHAP values can reveal which features with higher SHAP values for “Adnexal Torsion or Appendicitis”, play a more significant role in distinguishing between the two conditions. This information is valuable for feature selection, model interpretability, and domain-specific insights, allowing us to make more informed decisions in healthcare or other domains where precise prediction and feature importance are critical.

## 4. Results

A total of 21 patients diagnosed with confirmed adnexal torsion (group 1) and 20 patients diagnosed with acute appendicitis (group 2) constituted the subjects for this study. In group 1, the average age was 10 ± 2.6 years, while in group 2, it was 9.8 ± 21.9 years. To investigate the potential divergence in ages between the two patient groups, a *t*-test was conducted, yielding a *p*-value greater than 0.05. This outcome suggests no statistically significant age difference between the adnexal torsion and appendicitis groups.

**Figure 1:**
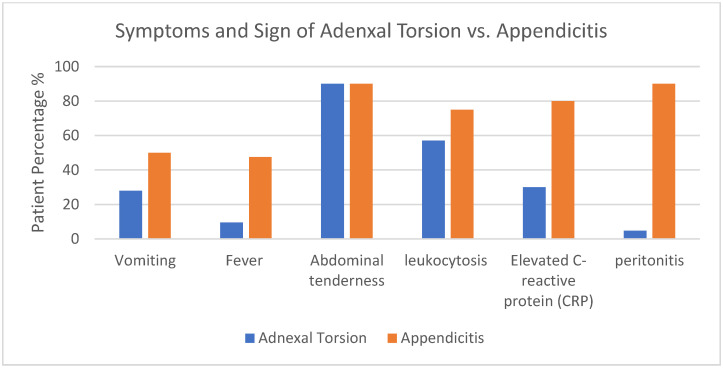
Symptoms and signs of adnexal torsion and appendicitis.

In terms of presenting symptoms, all patients experienced acute lower abdominal pain; however, group 1 displayed shorter pain duration (28.9 h vs. 46.8 h, *p* < 0.05), less vomiting (28% vs. 50%, *p* < 0.05), and lower fever incidence (4.7% vs. 50%, *p* < 0.05), compared to group 2. Over 90% of patients in both categories exhibited abdominal tenderness, although signs of localized peritonitis were identified in only one adnexal torsion patient, in contrast to the majority of acute appendicitis cases. Elevated white blood cell counts were observed in 57% in group 1 and 75% in group 2 (*p* < 0.05). Additionally, elevated C-reactive protein (CRP) levels were present in only 30% of group 1 compared to 80% of group 2 (*p* < 0.05). The distinctions in these symptoms and signs between adnexal torsion and appendicitis are depicted in Figure 1 and Table 3. 

Every patient with adnexal torsion and appendicitis underwent an ultrasound with color Doppler examination. In adnexal torsion cases, ultrasound findings indicated primary ovarian torsion characterized by an enlarged ovary positioned near the midline. This was associated with hyperechoic parenchyma and peripherally displaced follicles. Notably, the presence of an ovarian cyst was noted in 85% of adnexal torsion cases. Nevertheless, arterial flow, as determined by Doppler assessment, remained intact in all patients. For appendicitis patients, ultrasound findings encompassed two cases of a perforated appendix (10%) and 90% with acute uncomplicated appendicitis.

As previously discussed, we employed support vector classifier (SVC) functions, considering four crucial features, namely clinical presentation, pain duration, white blood cell counts, and ultrasound findings. Through rigorous cross-validation, our classifier demonstrated remarkable predictive accuracy in identifying adnexal torsion, with results spanning from 87% to 97%. These findings were bolstered by confidence intervals, which provide a measure of uncertainty in our estimates. The confidence intervals (0.69–1.05 and 0.83–1.07) indicate that, with a specified level of confidence, the true predictive accuracy of our SVC model falls within these ranges. It is important to note that the intervals extend beyond 100%, which could be attributed to the relatively small dataset used for training and validation, as detailed in Table 4.

Furthermore, we compared the SVM results with those of logistic regression and XGBoost models. While SVM with a polynomial kernel and logistic regression yielded favorable outcomes, logistic regression displayed wider confidence intervals, signifying a more cautious modeling approach. On the other hand, a narrower CI indicates more confidence and less variability in the model’s predictions. Of note, XGBoost consistently obtained flawless metrics on our modest dataset, which prompts concerns regarding the risk of overfitting. Even after implementing regularization, it maintained impeccable scores, hinting at the necessity for additional parameter adjustments. Therefore, we should exercise caution when solely depending on XGBoost’s exceptional performance, especially given our dataset’s limited size.

**Figure 2:**
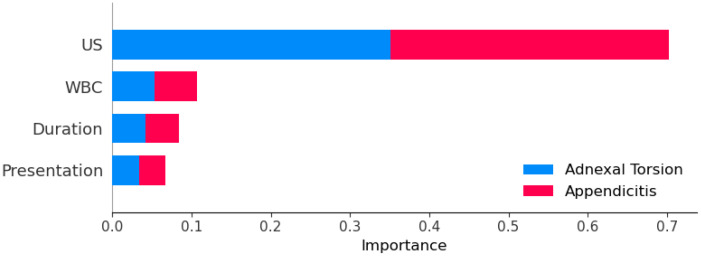
SHAP feature importance plot using the SVC with polynomial kernel.

In our assessment of feature significance using SHAP values for the categorization of adnexal torsion and appendicitis, we identified ultrasound (US) findings as the most influential feature, possessing the highest mean absolute SHAP value of 0.4461. In close succession, white blood cell (WBC) count demonstrated considerable importance, exhibiting a mean absolute SHAP value of 0.4720. Duration of pain, despite having a relatively elevated mean absolute SHAP value of 0.4705, held a slightly lower position in terms of importance when compared to US and WBC. Conversely, presentation had the lowest mean absolute SHAP value of 0.4810, signifying its limited impact on the model’s predictions. It is important to note that SHAP values represent the contribution of each feature to individual predictions. In contrast, the SHAP importance plot values, as visually depicted in Figure 2, provide an overview of the relative importance of features across the entire dataset. While SHAP values offer insight into how each feature influences individual predictions, the SHAP importance plot values help us understand the overall importance of features in the model’s decision-making process. These observations underline the significance of ultrasound and white blood cell count as pivotal factors for distinguishing between adnexal torsion and appendicitis within the SVM and polynomial kernel classification model, as supported by both SHAP values and the SHAP importance plot.

**Figure 3:**
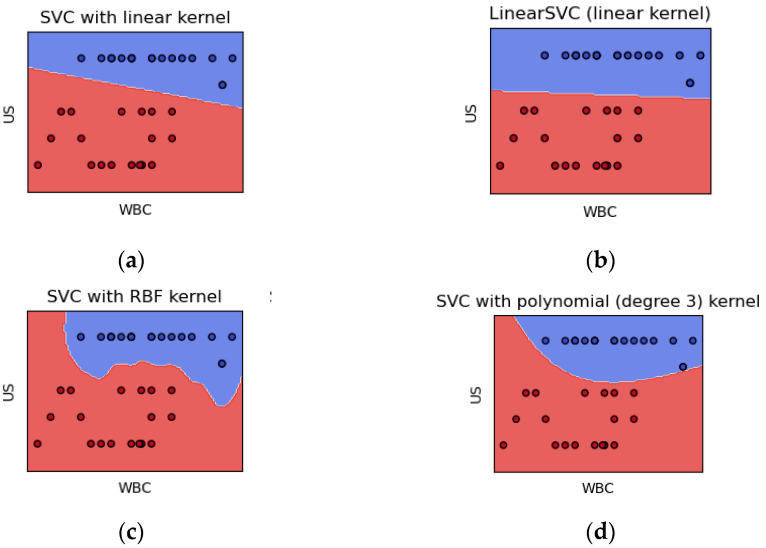
Representation of hyperplane and data point distribution for depicting the differentiation between patients with adnexal torsion (blue area) and patients with appendicitis (red area) in the hyperplane plot for SVM: (**a**) distribution of data points for support vector classifier (SVC) with linear kernel; (**b**) distribution of data points for linear SVC with linear kernel; (**c**) distribution of data points for SVC with radial basis function (RBF) kernel; (**d**) distribution of data points for SVC with polynomial kernel.

The visual representation of SVM’s performance is depicted in Figure 3, demonstrating the hyperplane plot. This plot effectively illustrates the differentiation between patients with adnexal torsion (depicted in the blue area) and those with appendicitis (represented in the red area). Notably, while the features of white blood cell count and ultrasound findings exhibited significant differences between the two conditions (*p* < 0.05), it is important to emphasize that these are just a part of the broader set of features analyzed. The results underscore the potential of machine learning techniques, particularly SVM, in enhancing diagnostic accuracy for distinguishing between cases of adnexal torsion and acute appendicitis. It is worth noting that the significance of other features was also assessed, contributing to the overall effectiveness of the SVM model in this critical differentiation task.

## 5. Discussion

The study investigates the application of machine learning in general, and specifically support vector machine (SVM) techniques, to improve the diagnosis of pediatric adnexal torsion, a condition often mistaken for acute appendicitis due to their similar symptoms. Many incidents have been reported of intraoperative finding of adnexal torsion in presumptive diagnosis of acute appendicitis [15], even when radiological studies such as ultrasound and CT scan were performed preoperatively [16].

Prior research in the field has examined the use of machine learning in the context of diagnosing adnexal torsion (AT) among pediatric patients [13]. While some studies have explored the potential of machine learning, it is essential to highlight that none of the existing literature specifically addressed the application of support vector machine (SVM) algorithms for these cases or employed the same combination of clinical presentation and ultrasound findings as predictive factors [11,12,13].

The research focuses on analyzing four key clinical features: clinical presentation, pain duration, white blood cell counts, and ultrasound findings. The SVM model, with various kernel functions, shows promise in enhancing diagnostic accuracy, with the polynomial kernel function exhibiting the most favorable performance.

The current body of scholarly work consists of investigations that have assessed various machine learning techniques for the diagnosis of adnexal torsion in pediatric populations. This distinction sets our research apart, as it concentrates on assessing the capability of SVM, a widely recognized machine learning method, to differentiate adnexal torsion from acute appendicitis using a unique combination of clinical features.

Our study aligns with the broader trend in current research, which highlights the advantages of machine learning approaches in the healthcare domain [13]. More specifically, it emphasizes how these proposed machine learning algorithms can be seamlessly integrated into Medical Decision Support Systems (MDSSs) to assist healthcare practitioners in their decision-making processes [13].

It is worth acknowledging that SVM, known for its classification prowess, has demonstrated its effectiveness even when working with relatively limited datasets, as highlighted in previous research [17]. This aspect holds particular significance in our study, as it contributes to the practicality of implementing SVM in real-world clinical scenarios where data availability may be constrained.

Despite the promising results, the study has several limitations. First, the dataset used in the study is relatively small, which may introduce bias and limit the generalizability of the findings. Additionally, the study lacks external validation, which is crucial to assess the model’s performance on different patient populations. Moreover, the study focuses on a single medical center, which may not represent diverse patient demographics or healthcare settings. Lastly, while the SVM model shows potential, the clinical implementation of such technology requires user-friendly tools and further validation in larger cohorts.

The study has significant implications for medical practice, policy, and future research. From a practical perspective, the findings suggest that SVM, mainly when using the polynomial kernel function, can aid in the early and accurate diagnosis of adnexal torsion in pediatric patients. From a policy standpoint, the potential of integrating the SVM algorithm into Medical Decision Support Systems (MDSSs) and health portal systems should not be underestimated. These systems can serve as valuable tools to assist healthcare providers in their decision-making processes, providing them with evidence-based recommendations and enhancing diagnostic accuracy. For future research, there is a need for more extensive multicenter studies to validate the findings and assess the model’s performance in diverse patient populations.

## 6. Conclusions

In conclusion, this study offers promising insights into the potential of machine learning techniques, particularly support vector machines (SVMs), to enhance the diagnosis of pediatric adnexal torsion. While acknowledging the study’s limitations, including its small dataset and the imperative for external validation, the findings highlight the promise of SVM, particularly when employing the polynomial kernel function, in elevating diagnostic precision, especially in cases where adnexal torsion manifests symptoms akin to acute appendicitis. Additionally, it is essential to explore and validate other machine learning approaches, especially when armed with more extensive datasets, as they hold the promise of delivering even more robust decision-making capabilities. This research contributes to the ongoing endeavors to harness technology for more precise and informed medical decisions, ultimately benefiting patient care and outcomes. To fully unlock the clinical potential of machine learning in this domain, additional research and validation are imperative.

## Figures and Tables

**Table 1:** Adnexal torsion patients’ symptoms.

Subject	Age	Presentation ^1^	Duration (Hours)	WBC ^2^	Ultrasound ^3^	Lateralization	Mass
1	10	1	48	12	1	left	yes
2	11	1	72	10	1	right	yes
3	2	1	5.04	13	1	left	yes
4	7	2	6	16	2	right	no
5	8	1	7.2	14	2	left	yes
6	9	1	16.8	4	2	right	no
7	10	2	12	7	2	right	no
8	11	2	24	6	3	left	yes
9	12	1	48	14	1	right	yes
10	14	1	18	2.7	1	right	yes
11	9	1	16.8	9	1	right	yes
12	10	1	21.6	5	3	right	yes
13	8	1	24	13	1	right	yes
14	12	1	48	14	1	right	yes
15	10	1	24	16	1	right	yes
16	11	1	24	11	1	left	yes
17	10	1	48	13	3	right	yes
18	12	2	24	8	1	left	yes
19	13	2	72	12.8	1	right	yes
20	8	1	24	14	1	left	yes
21	13	2	24	9.6	1	left	Yes

^1^ Presentation Assigned Numeric Value, as inserted into the SVM classifier, 1: pain; 2: pain and vomiting; ^2^ White blood cell counts; ^3^ Ultrasound Assigned Numeric Value, as inserted into the SVM classifier, 1: ovarian cyst; 2: enlarged ovary/peripheral follicles; 3: adnexal mass.

**Table 2:** Appendicitis patients’ symptoms.

Subject	Age	Presentation ^1^	Duration (Hours)	WBC ^2^	Ultrasound ^3^
1	7	3	72	21	4
2	7	1	24	22	5
3	9	3	48	12	5
4	12	1	48	14	5
5	9	3	120	21	4
6	12	1	24	15	5
7	12	3	72	17	5
8	12	5	48	14	5
9	9	1	24	9	5
10	11	1	24	11	5
11	6	1	48	12	5
12	12	3	72	7	5
13	12	3	48	16	5
14	8	3	72	10	5
15	12	1	24	10	5
16	10	4	24	20	5
17	9	5	48	10	5
18	8	2	24	12	5
19	9	2	24	12	5
20	10	3	48	18	5

^1^ Presentation Assigned Numeric Value, as inserted into the SVM classifier, 1: pain; 2: pain and vomiting; 3: pain, vomiting and fever; 4: pain and nausea; 5: pain and fever; ^2^ White blood cell counts; ^3^ Ultrasound Assigned Numeric Value, as inserted into the SVM classifier, 4: perforated appendix; 5: acute appendicitis.

**Table 3:** Demographic and clinical data for all groups.

Variable	Adnexal Torsion (*n* = 21)	Acute Appendicitis (*n* = 20)	*p*-Value ^1^ (α = 0.05)
Mean Age	10 ± 2.6	9.8 ± 1.9	>0.05
Pain Duration (hour)	28.9	46.8	<0.05
Vomiting	28.5%	50%	<0.05
Fever	4.7%	50%	<0.05
Leukocytes	57%	75%	<0.05
CRP	30%	80%	<0.05

^1^ *t*-test *p*-value results for the comparison of the mean difference between (group 1) and (group 2).

**Table 4:** Performance of SVC functions.

SVC Metric	SVC Function ^1^			Logistic Reg. 95% CI ^2^
	Polynomial 95% CI ^2^	Linear 95% CI ^2^	Radial Basis 95% CI ^2^	
Accuracy	97.0% 0.83–1.07	92.0% 0.79–1.05	87.0% 0.69–1.05	97.3% 0.73–1.21
AUC ^3^	95.0% 1.00–1.00	99.0% 1.00–1.00	92.5% 0.92–1.04	97.2% 0.72–1.22
Precision Score	93.0% 0.76–1.09	91.0% 0.73–1.12	91.0% 0.55–1.25	100% 1.00–1.00
Recall Score	99.0% 1.00–1.00	96.0% 0.81–1.08	95.0% 0.70–1.08	94.4% 0.44–1.44
F1-Score	96.0% 0.86–1.05	94.0% 0.81–1.04	93.0% 0.78–0.98	97.0% 0.73–1.23

^1^ Support vector classifier functions; ^2^ 95%Confidance Interval; ^3^ Area under the curve.

## Data Availability

The data presented in this study are available on request from the corresponding author. The data are not publicly available due to privacy reasons.
